# Glucocerebrosidase mRNA is Diminished in Brain of Lewy Body Diseases and Changes with Disease Progression in Blood

**DOI:** 10.14336/AD.2017.0505

**Published:** 2018-04-01

**Authors:** Laia Perez-Roca, Cristina Adame-Castillo, Jaume Campdelacreu, Lourdes Ispierto, Dolores Vilas, Ramon Rene, Ramiro Alvarez, Jordi Gascon-Bayarri, Maria A. Serrano-Munoz, Aurelio Ariza, Katrin Beyer

**Affiliations:** ^1^Department of Pathology, Hospital Universitari and Health Sciences Research Institute Germans Trias i Pujol, Universitat Autònoma de Barcelona, Spain; ^2^Department of Neurology, Hospital Universitari de Bellvitge, L’Hospitalet de Llobregat, Spain; ^3^Department of Neurology, Hospital Universitari Germans Trias i Pujol, Badalona, Barcelona, Spain.

**Keywords:** glucocerebrosidase deficiency, Parkinson’s disease, dementia with Lewy bodies, GBA mRNA expression, transcript variants

## Abstract

Parkinson disease (PD) and dementia with Lewy bodies (DLB) are Lewy body diseases characterized by abnormal alpha-synuclein deposits and overlapping pathological features in the brain. Several studies have shown that glucocerebrosidase (GBA) deficiency is involved in the development of LB diseases. Here, we aimed to find out if this deficiency starts at the transcriptional level, also involves alternative splicing, and if GBA expression changes in brain are also detectable in blood of patients with LB diseases. The expression of three *GBA* transcript variants (GBAtv1, GBAtv2 and GBAtv5) was analyzed in samples from 20 DLB, 25 PD and 17 control brains and in blood of 20 DLB, 26 PD patients and 17 unaffected individuals. Relative mRNA expression was determined by real-time PCR. Expression changes were evaluated by the ΔΔCt method. In brain, specific expression profiles were identified in the temporal cortex of DLB and in the caudate nucleus of PD. In blood, significant GBA mRNA diminution was found in both DLB and PD patients. Early PD and early-onset DLB patients showed lowest GBA levels which were normal in PD patients with advanced disease and DLB patients who developed disease after 70 years of age. In conclusion, disease group specific GBA expression profiles were found in mostly affected areas of LBD. In blood, GBA expression was diminished in LB diseases, especially in patients with early onset DLB and in patients with early PD. Age of disease onset exerts an opposite effect on GBA expression in DLB and PD.

Parkinson disease (PD) and dementia with Lewy bodies (DLB) belong to the group of Lewy body diseases (LBDs) and are characterized by abnormal aggregates of alpha-synuclein, so called Lewy body pathology (LBP), in the brain [1]. Neuropathologically, two LBP forms can be distinguished: pure LBP, showing LBP only, and common LBP, with a mixture of LBP and concomitant Alzheimer disease (AD) pathology [2]. Lewy bodies are intraneuronal proteinaceous inclusions containing as a main component. They are found in the substantia nigra and other brain stem nuclei in early PD [3] and throughout almost all brain areas in DLB [4]. Usually, about 20%-50% of PD patients develop dementia (PDD) after no less than 10 to 15 years following PD diagnosis [5].

Glucocerebrosidase (GCase) is a lysosomal enzyme responsible for the breakdown of glucocerebroside into glucose and ceramide [6]. Mutations in the GCase gene *GBA* cause GCase deficiency leading to glucocerebroside accumulation inside the lysosome. This accumulation results in Gaucher disease, the most frequent lysosomal storage disorder [6]. Since several probands with Gaucher disease present parkinsonism [7] and have *GBA* mutation-carrier relatives with PD [8], subsequent studies have revealed that *GBA* mutations are strongly associated with PD but also with DLB [9-11].

Analyses of GCase activity and expression levels in PD brains have shown that GCase activity and protein levels are diminished in sporadic PD with and without *GBA* mutations [12, 13]. Furthermore, decreased GCase activity has been found in blood of PD patients [14]. Decrease of GCase activity causes the accumulation of glucocerebroside in lysosomes, directly promoting AS oligomerization and fibrillation. At the same time, AS fibrils inhibit GCase activity creating a bidirectional pathogenic loop [15].

Over the past years, deregulation of alternative splicing has been described repeatedly as an important mechanism involved in ageing and disease development [16, 17]. In this context, we have reported that differential isoform expression changes are involved in LBD pathogenesis [18, 19]. For the GBA gene, five transcript variants (tv; http://www.ncbi.nlm.nih.gov/gene/2629) have been reported by the NCBI database. GBAtv1-3 are the result of alternative inclusion of their initial exons encoding the same protein. GBAtv4 and tv5 are the result of splicing out of exons 2 and 3 or exon 5, and bear shorter proteins.

In this study, we addressed three main questions. First, we wanted to know if GCase deficiency in LBD starts at the transcriptional level; second, if possible brain GBA expression changes are also detectable in blood of LBD patients and third, if alternative *GBA* splicing is dysregulated in these patients.

## MATERIALS AND METHODS

### Brain tissues

Post-mortem brain samples and their corresponding clinical and neuropathological diagnoses were provided by the Institute of Neuropathology Brain Bank and the Neurological Tissue Bank of the University of Barcelona / Hospital Clinic, Barcelona, Spain. They were obtained from 20 patients with clinical diagnosis of DLB, 25 patients with clinical diagnosis of PD, and 17 donors devoid of neurological signs or symptoms and lack of neuropathological findings. Eight of the DLB brains did not present AD-related pathology and were defined as pure DLB (pDLB), while 12 DLB brains contained concomitant AD-related pathology and were considered as common DLB (cDLB). Of the 25 PD patients, 13 developed dementia (Parkinson’s disease with dementia; PDD) but 12 did not (Parkinson’s disease without dementia; PDND). None of the patients included in this study carried GBA mutations. Neuropathological diagnosis was carried out as described before [20]. Two brain areas, temporal cortex and caudate nucleus, were analyzed for all disease groups, and the pons was available for PD only. Frontal cortex samples were used to estimate relative expression levels of GBA transcripts. Clinical and neuropathological characteristics of patients and controls are summarized in Table 1.

**Table 1:** Clinico-neuropathological characteristics of Lewy body disease cases and controls.

Disease	n	PMtime^1^ (range)	ADstage^2^	Br&Br^3^	Death^4^ (range)	M:F ratio^5^
pDLB^6^	8	9:30 (3:30-17:00)	0-II	A-C	74.6 (60-85)	3:1
cDLB^7^	12	10:30 (4:00-21:15)	III-VI	B-C	79.0 (74-86)	1.4:1
PD^8^	12	7:00 (3:30-14:00)	III-IV		80.8 (68-93)	1:1
PDD^9^	13	7:10 (4:00-12:20)	II-VI	A-C	78.7 (71-87)	0.9:1
CTRL^10^	17	8:40 (2:30-23:30)			69.3 (55-81)	1.4:1

1 post-mortem time;

2 AD stages following Braak and Braak, I-VI: neurofibrillary tangles;

3 AD stages following Braak and Braak, A-C: amyloid plaques;

4 death, age at death;

5 M:F ratio, male-female ratio;

6 pDLB, dementia with Lewy bodies, pure form;

7 cDLB, common dementia with Lewy bodies;

8 PD, Parkinson disease without dementia;

9 PDD, Parkinson disease with dementia;

10 CTRL, control brain samples.

### Patients

Twenty DLB patients (mean age, 73.9; mean age of onset, 68; mean disease duration, 5.9 years; male-female ratio, 1:0.5) were recruited by the Department of Neurology of the Bellvitge Hospital and were diagnosed according to the 2005 DLB Consortium criteria [21]. Twenty-six PD patients (mean age, 68.9; mean age of onset, 65.3; mean disease duration, 6.5 years; male-female ratio, 1:1.4) were diagnosed in the Department of Neurology of the Hospital Germans Trias i Pujol following UK Parkinson’s disease Brain Bank clinical diagnostic criteria [22]. None of them presented dementia when blood samples were obtained, and no GBA mutation carriers had been identified. Age at onset was defined as the age when memory loss or parkinsonism was first noticed by relatives. Seventeen control individuals (mean age, 74.4; male-female ratio, 1:1.1) were devoid of neurological symptoms and familial history of neurodegenerative disease and were recruited by both Neurology departments. Written informed consent was obtained from all subjects, either directly or from their legal guardians. The study was carried out with the approval of our local Ethics Committee for Clinical Investigation.

### RNA isolation, reverse transcription and assessment of mRNA stability

TRI-Reagent (MRC, Cincinnati, USA) was used for RNA isolation according to the manufacturer’s protocol. RNA quantity, purity and integrity was ascertained by the Agilent 2100 Bioanalyzer (Agilent Technologies, Santa Clara, USA). Only samples with RIN values higher than 6 were stored at -80°C until use. Two μg of total RNA were used for reverse transcription by Ready-to-go™ You-Prime First-Strand Beads (GE Healthcare, Buckinghamshire, UK). A “no RT” reaction, using water instead of RT, served as a control for the exclusion of genomic DNA contamination. cDNAs were either used immediately for PCR amplification or stored at -20ºC.

Stability assessment of the transcripts analyzed in this study was carried out by defining their degradation rates at RIN values of about 6 [20].

**Table 2:** RNA primer sequences used for the amplification of *GBA1* isoforms, beta-actin, *GUS* and *PBGD*.

Name and NCBI^1^	Primer name	Primer sequence (5’ - 3’)	Size^2^
GBA1tv1 NM_000157.3	GBA1tv1U^*^	ATC ACA TGA CCC ATC CAC A	214 bp^3^
	GBA1tv1L	ACT CAA AGG CTT GGG ACA T	
GBA1tv2 NM_001005741.2	GBA1tv2U2^*^	TTC GCC GAC GTG GAT CCT CT	236 bp
	GBA1tv2L2	ACC GAG CTG TAG CCG AAG CT	
GBA1tv3 NM_001005742.2	GBA1tv3U^*^	TTC GCC GAC GAG ACT CTG GA	176 bp
	GBA1tv3L	ACC TGA TGC CCA CGA CAC TG	
GBA1tv4 NM_001171811.1	GBA1tv4U^*^	TTC TCT TCG CCG ACG GTG CC	169 bp
	GBA1tv4L	AGC TCC ATC CGT CGC CCA CT	
GBA1tv5 NM_001171812.1	GBA1tv5U^*^	ACG GGC ACA GGA ATC GGA TA	173 bp
	GBA1tv5L	AAC TGC AGG GCT CGG TGA AT	
	b-act U2	TCT ACA ATG AGC TGC GTG TG	228 bp^4^
	b-act L2	GGA TAG CAA CGT ACA TGG CT	
	b-act U3	AAC TGG GAC GAC ATG GAG AA	178 bp^5^
	b-act L3	TAG ATG GGC ACA GTG TGG GT	
	GUS^6^-U1	ATG TGG TTG GAG AGC TCA TT	176 bp
	GUS-L2	TGT CTC TGC CGA GTG AAG AT	
	PBGD^7^_U1	ACA CAC AGC CTA CTT TCC AAG	183 bp
	PBGD_L1	TCA ATG TTG CCA CCA CAC TGT	

1 Name and NCBI, name of the transcript variant and NCBI accession number;

2 Size, amplicon size;

3 bp, base pairs;

4 228 bp, amplicon size resulting from primer pair b-actU2 + b-actL3;

5 178 bp, amplicon size resulting from primer pair b-actU3 + b-actL2;

6 GUS, beta-glucuronidase;

7 PBGD, porphobilinogen deaminase.

* These primers comprise transcript-specific sequences.

**Figure 1.**
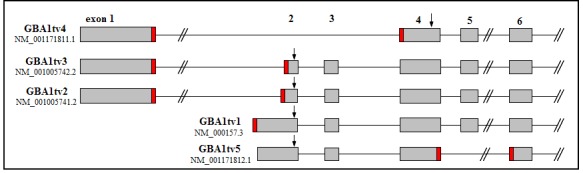
Schematic representation of the five *GBA* transcripts and location of forward primers. Grey boxes represent exons and the lines, introns. Narrow red rectangles at the end of some exons indicate sequences, chosen for designing isoform-specific primers.

### Primer design

Primers were designed for specific amplificacition of *GBA* transcripts. Primer location is shown in Figure 1, and sequences and amplimer sizes in Table 2. The *GBA* gene bears 5 transcript variants by the inclusion of alternative 5′ initial exons and by splicing out of internal exons. All five show specific sequences (www.ncbi.nlm.nih.gov/gene/2629) permitting the design of isoform-specific primers. The design of primers for the *GBA* gene is especially challanging because of its high homology with the *GBAP1* gene [23]. Therefore not all primers could be designed to rise 150-180 bp long amplicons (Table 2). To amplify GBAtv1, the forward primer was located in exon 2 and the reverse primer in exon 3 (Fig. 1). Since GBAtv5 lacks its exon 4, the forward primer spanned the boundary between exons 4 and 6, and the reverse primer was located in exon 7. Transcripts GBAtv2 and GBAtv3 have different initial sequences of their exons 2, while GBAtv4 lacks exons 2 and 3, differences that were used to design specific primers (Fig. 1).

### Assessment of SNCB mRNA stability

High quality RNA cannot be taken for granted when dealing with post-mortem brain samples, especially with post-mortem times larger than 5 hours [24]. In order to assure that *GBA*, *ACTB, GUSB* and *PBGD* RNAs presented similar stabilities even at RIN values of about 6, we assessed their RNA degradation rates [25]. Ten µg of RNA corresponding to two of each, temporal cortices and caudate nucleus, were incubated at 50°C. Two-µg-aliquotes were withdrawn from each sample after 15, 30, 60, 120, and 240 minutes, respectively. Of these, 1 µg was subjected to the study of RNA integrity using the Agilent 2100 Bioanalyzer and from 1 µg cDNA was obtained. Incubation time dependent RNA degradation coincided with diminishing RIN values. Real-time PCR analysis of the relative amount of the three GBA transcripts showed that none of the GBA transcripts degraded at higher rates than *ACTB, GUSB* and *PBGD* mRNAs, or vice versa.

### Real time PCR

Relative expression was determined for three GBA transcript variants (GBAtv1, GBAtv2 and GBAtv5). Real-time PCR was carried out on a Rotor-Gene 6000 (Corbett Life Science, Sydney, Australia). PCR was performed in 15 µl reactions with the QuantiTect SYBR Green PCR Kit (QiaGen, Hilden, Germany), containing 16 pmol of each primer and 1 µl of cDNA. To estimate relative expression changes, two housekeeping genes were analyzed in each brain region, beta-actin (*ACTB*) and beta-glucuronidase (*GUSB*) [26], and two housekeeping genes were analyzed in blood as well, *ACTB* and porphobilinogen deaminase (*PBGD*) [27]. Primer sequences are listed in Table 2.

All assays included two replicates of each sample, were performed twice and independently to assure their reproducibility and minimize possible errors. Standard curves for the target and reference genes were generated for each run by amplifying the same serially diluted cDNA control sample [28, 29].

To assess relative gene expression, relative transcript variant expression data were obtained by the ΔΔCt method based on similar PCR efficiencies to analyze relative gene expression [26, 27].

Differences among GBAtv1, GBAtv2 and GBAtv5 expression levels, were calculated by 2^n, where n corresponds to the cycle number difference between Ct of one of the less expressing transcript and Ct of the major transcript [30].

### Statistical analysis

Analyses were performed independently for both housekeeping genes and the mean obtained from both analyses represented the final expression change of *GBA* isoforms compared to controls for which expression level is assumed to be 1. In the text, all values are given as mean values with variance estimates in brackets. According to the ΔΔCt method, values below 0.5 represented significantly decreased expression levels, while values above 1.5 corresponded to significantly increased expression levels. Nevertheless, results were accepted as significant only with 0.5 as major variance value in the case of expression decrease and with 1.5 as minor variance value in the case of expression increase. To take into account the exponential function of Ct values, in the final calculation the variance was estimated by evaluating 2^-ΔΔCt^ term using ΔΔCt plus the standard deviation and ΔΔCt minus the standard deviation [29].

Differences between age, disease onset and disease duration were assessed by t-test, and regression analyses was performed to evaluate the possible association between GBAtv1 expression levels and age, disease onset or disease duration. Statistical analyses were performed using the SPSS 21 (IBM, Armonk, NY, USA) software, and Statpages (http://www.statpages.org).

**Figure 2.**
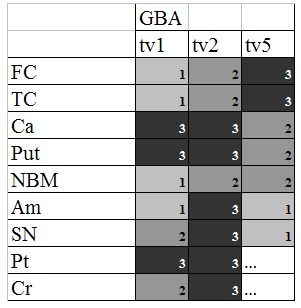
Relative *GBA* isoform expression in different brain areas. *GBA* expression in neural tissue estimated by appraising agarose gel electrophoretograms: tv, transcript variant; FC, frontal cortex; TC, temporal cortex; Ca, caudate nucleus; Put, putamen; NBM, Nucleus basalis of Meynert; Am, Amygdala; SN, Substantia nigra; Pt, pons; Cr, cerebellum. White fields correspond to lack of expression, light gray (1) to very slight expression, middle gray (2) to readily detectable expression, and dark gray (3) to high expression. The black fields represent very intense expression levels.

### RESULTS

Of the five GBA transcripts, GBAtv3 and GBAtv4 showed very low expression in brain as well as in blood. Therefore, it was not possible to analyze their relative expression.

### GBA transcripts expression in different brain areas

Analysis of *GBA* isoform expression in different brain areas revealed highest *GBA* levels in the caudate nucleus and putamen, with strong GBAtv1 and tv2 expression and intermediate GBAtv5 expression. In contrast, both cortical areas, frontal and temporal cortices, showed only low or intermediate GBAtv1 and tv2 expression, but strong GBAtv5 expression (Fig. 2). GBAtv1 was mostly expressed in the caudate nucleus, putamen and pons, as compared with the other brain areas analyzed. GBAtv2 showed almost uniformly high expression in all brain areas studied. On the contrary, GBAtv5 was expressed mostly in the cortex and showed only traces of expression in the pons and cerebellum (Fig. 2).

### GBA isoform mRNA expression changes in the temporal cortex, caudate nucleus and pons from LBD brains

All results are represented as relative expression changes compared to normal control brain areas with variance estimations in brackets. Only significant changes are shown in the text.

In the temporal cortex, GBAtv1 expression was diminished in pDLB (0.38 (0.29-0.49)), and in cDLB (0.27 (0.14-0.49)), but unchanged in both PDND and PDD. GBAtv2 did not show significant expression changes in any of the groups. GBAtv5 was almost 4-fold decreased in pDLB (0.35 (0.23-0.49)), but not in the other groups.

In the caudate nucleus, GBAtv1 was significantly diminished in PDD (0.41 (0.38-0.45)), pDLB - 0.41 (0.30-0.58) and cDLB - 0.44 (0.34-0.61). No changes in GBAtv1 expression were observed in PDND. GBAtv2 expression did not change in any of the groups, and GBAtv5 was diminished only in PDD (0.36 (0.34-0.37)).

Samples of pons were only available for PD, PDD and control brains. None of the GBA isoforms was altered in PDND or PDD in this brain area (data not shown).

**Figure 3.**
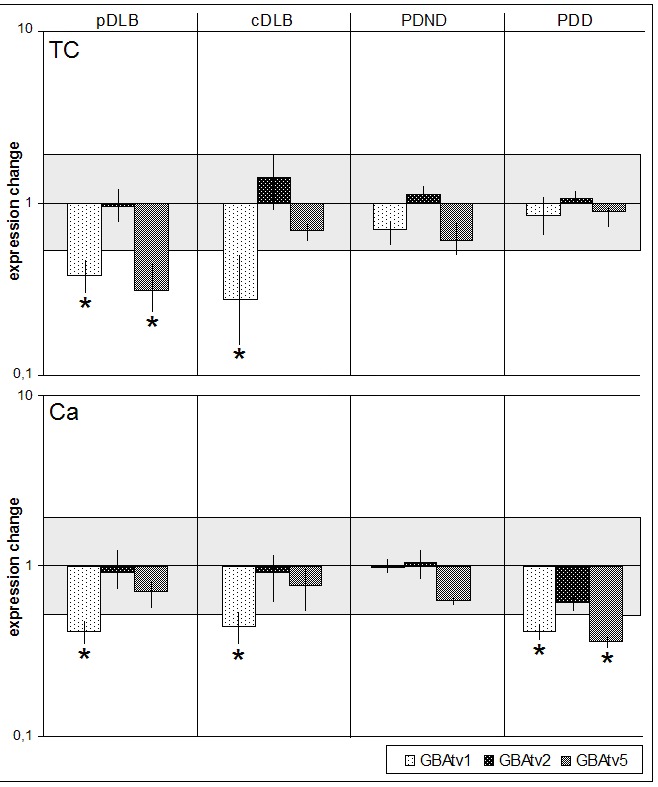
Expression profiles of *GBA1* isoforms in three brain areas of LBD after adjustment with controls. The included areas were temporal cortex (TC) and caudate nucleus (Ca) from the groups of pure dementia with Lewy bodies (pDLB), common dementia with Lewy bodies (cDLB), Parkinson′s disease without dementia (PDND) and Parkinson′s disease with dementia (PDD). The results are shown as relative expression changes obtained by the ΔΔCt method in comparison with normal controls and are represented in a logarithmic scale. Grey areas represent normal expression range. * Significant expression change below 0.5.

Expression profiles (Fig. 3) revealed the presence of disease-specific and brain-area specific expression changes of *GBA* isoforms. Disease-specific expression profiles were detected for pDLB and cDLB in the temporal cortex. Both, PD with and without dementia showed overlapping expression profiles in the temporal cortex. In contrast, both DLB groups showed overlapping expression profiles in the caudate nucleus. On the contrary, PD groups showed differential expression profiles in this brain area (Fig. 3).

### Characterization of patients by onset, duration and disease progression indicators

The influence of age at onset and disease duration on GBA expression levels in blood was studied by dividing patients into the following groups: 1. according to age at onset: (a) patients who developed the disease at age of 65 years or before and (b) patients who developed disease at the age of 66 years or later; and 2. according to the duration of disease: (a) less than 6 years or (b) 6 years or more. Table 3 shows that disease duration from onset was similar in both age-at-onset dependent DLB groups. On the contrary, in PD disease duration from onset was significantly longer in patients who developed PD at the age of 65 years or earlier when compared to patients with PD onset after 65 years.

**Figure 4.**
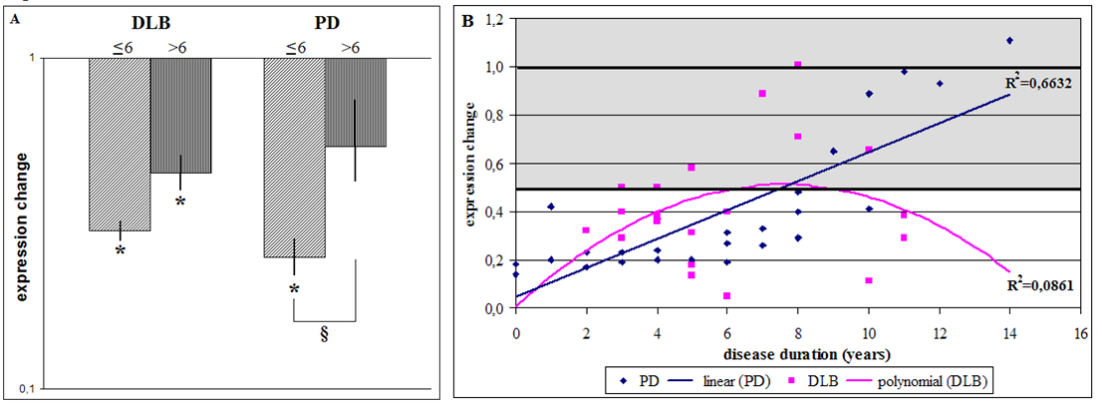
GBA1tv1 expression in blood of DLB and PD patients in dependency on disease duration. GBA1tv1 expression was analyzed (A) in two groups and (B) for each patient individually. For (A), the results are shown as relative expression changes obtained by the ΔΔCt method in comparison with control individuals. * Significant expression change below 0.5. § Significant expression change between the disease duration subgroups. For (B) each point corresponds to the value of the expression change of each individual obtained by the ΔΔCt method, where ΔCt of patients was determined individually and ΔCt of control individuals was the mean value of the entire control group. Grey areas represent normal expression range.

### GBA mRNA expression in blood

Differential expression analysis of the three *GBA* transcripts in blood of DLB and PD patients unveiled that GBAtv1 was significantly decreased in DLB (0.41 (0.38-0.44)) and PD (0.35 (0.32-0.39)). In contrast, neither GBAtv2 nor GBAtv5 showed significant expression changes in blood of DLB or PD patients (data not shown).

To further assess the possible association between GBA mRNA expression and clinical features, data were analyzed taking into account gender, disease duration from onset and age at disease onset. No differences in GBAtv1 expression levels were found for different gender.

### Expression of GBAtv1 and disease duration

Although there was a tendency of lower GBAtv1 expression (0.30 (0.29-0.32)) in blood of DLB patients with shorter disease duration from onset versus patients with longer disease duration (0.45 (0.42-0.50)), this difference (0.64 (0.58-0.73)) was not significant between both onset groups (Fig. 4A). In blood of PD patients in turn, patients with early PD showed two times less GBAtv1 expression levels (0.26 (0.21-0.32)) than patients with 6 years of disease duration or more, where GBAtv1 diminution was no longer significant (0.55 (0.43-0.71); Fig. 4A).

These differences between DLB and PD prompted us to analyze in depth the relation between disease duration and GBAtv1 expression in blood. As shown in Figure 4B, the effect of disease duration on GBAtv1 expression in blood was different in DLB and PD. Whereas in DLB the first years of disease were characterized by low GBAtv1 levels, GBAtv1 expression reached normal levels in patients with disease duration between 6 and 8 years and diminished again in DLB patients with longer disease duration. On the contrary, in PD a linear correlation between GBAtv1 expression and duration of disease was detected. Initial stages of the disease were characterized by most pronounced GBAtv1 diminution which levels raised to normal after about 10 years of disease progression (Fig. 4B).

### Expression of GBAtv1 and age at disease onset

**Figure 5.**
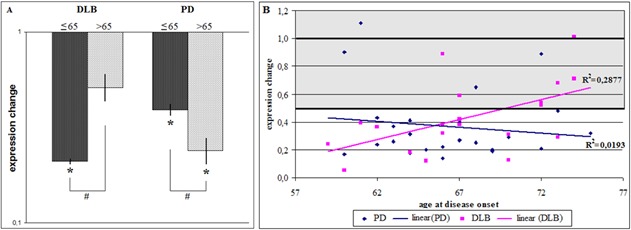
GBA1tv1 expression in blood of DLB and PD patients in dependency on the age of disease onset. GBA1tv1 expression was analyzed (A) in two groups and (B) for each patient individually. For (A), the results are shown as relative expression changes obtained by the ΔΔCt method in comparison with control individuals. *Significant expression change below 0.5. #Significant expression change between the age-at-onset dependent subgroups. For (B) each point corresponds to the value of the expression change of each individual obtained by the ΔΔCt method, where ΔCt of patients was determined individually and ΔCt of control individuals was the mean value of the entire control group. Grey areas represent normal expression range.

As shown in figure 4A, when compared to controls, GBAtv1 expression was five times lower in patients in blood of DLB patients with early onset (0.21 (0.20-0.22)) and almost two times lower than in those who started after age of 65 years (0.51 (0.44-0.60)). Interestingly, GBAtv1 expression was also more than two times lower in patients with early onset when compared to later onset DLB (0.39 (0.33-0.46)). On the contrary, in PD, compared to controls, patients with earlier disease onset had two times lower GBAtv1 expression (0.39 (0.36-0.41) while patients who developed PD after the age of 65 years had four-times lower GBAtv1 (0.24 (0.17-0.38)). Early onset PD had 2-times higher GBAtv1 expression than patients who developed PD after the age of 65 years (1.90 (1.54-2.66); Fig. 5A). Data were also plotted individually and underwent regression analysis. As shown in figure 4B, linear association between disease onset and GBAtv1 expression was observed for both DLB and PD, but with opposite effects. Whereas GBAtv1 expression was drastically diminished in DLB patients with early disease onset but was normal in patients of 72 years or older, PD patients who debuted before the age of 60 years presented normal GBAtv1 levels which were decreased in patients with later onset (Fig. 5B).

**Table 3:** Clinical characteristics of DLB and PD patients in the disease onset and duration groups.

DLB			
Disease onset	<65 years	>65 years	*P*^1^
n	6	14	
age at onset (range)	61.4 (59-65)	68.5 (66-74)	n.p.^2^
age (range)	67.6 (63-71)	73.8 (69-80)	n.p.
duration (range)	6.2 (2-10)	4.9 (2-10)	0.135
male: female ratio	1: 0.33	1: 0.4	0.765
Disease duration since onset	<6 years	>6 years	
n	13	7	
age at onset (range)	67.1 (59-74)	63.5 (60-67)	0.238
age (range)	70.8 (63-80)	72.8 (70-77)	0.302
male:female ratio	1: 0.5	1: 0.25	0.097
PD			
Disease onset	<65 years	>65 years	
n	12	14	
age at onset (range)	62.2 (60-64)	70.0 (68-73)	n.p.
age (range)	69.0 (61-75)	72.5 (68-75)	n.p.
duration (range)	6.8 (1-14)	2.5 (0-6)	0.015
male: female ratio	1: 0.8	1:01	0.827
Disease duration since onset	<6 years	>6 years	
n	15	11	
age at onset (range)	66.9 (60-73)	61.7 (60-64)	0.105
age (range)	69.6 (61-75)	72.3 (68-75)	0.376
male: female ratio	1: 0.9	1: 0.9	1

1 p-value, obtained by t-test;

2 n.p., does not proceed.

## DISCUSSION

### Expression of GBA transcripts in brain

In the present study we have analyzed the differential expression of three GBA transcript variants in the temporal cortex, caudate nucleus and pons of DLB and PD brains divided into pure and common DLB and into PD with and without dementia. Although tissue specific expression of alternative splice variants has been described earlier, it has been also shown that isoforms expression changes in different brain areas are associated with disease [31, 32]. Accordingly, we identified specific expression profiles in the temporal cortex of DLB with decreased GBAtv1 expression in cDLB and combined GBAtv1 and tv5 diminution in pDLB. The specific expression profile of GBA transcripts in PDD caudate nucleus also showed the decrease of both GBAtv1 and tv5. In the caudate nucleus, GBAtv1 was also diminished in both DLB groups. Although these results suggest a defined role for GBAtv5 during pathogenesis of LBD, the specific function of this minor isoform remains to be determined. Our findings furthermore underline the specific involvement of cortical regions in DLB [33] and the association of the caudate nucleus with dementia in LBD. In this context, it has been shown that dopamine depletion in the caudate nucleus can be detected in DLB [34] and correlates with cognition in PD [35].

In two recent studies GCase activity and expression levels were analyzed in PD brains. Whereas Gegg and colleagues analyzed cerebellum, frontal cortex, putamen, amygdala and substantia nigra samples from patients with sporadic PD and from patients with *GBA* mutations [36], Murphy and colleagues investigated anterior cingulate cortex and occipital cortex in early and late PD stages [12]. Results revealed diminished GCase activity and protein levels in cerebellum, substantia nigra [36] and anterior cingulate cortex, a region that accumulates abnormal AS in sporadic PD [12] and additionally in putamen and amygdala of PD brains with *GBA* mutations [36]. In both studies, no changes in GCase levels or activity were found in the cortical areas. Both studies further coincided in no finding any significant GBA mRNA expression changes, indicating that in PD GCase expression changes occur at the posttranscriptional level. Our results are in concordance with those of Gegg’s study showing unchanged GBA mRNA expression levels in the cortex of PD patients with and without dementia. In DLB, GCase protein levels have been found to be diminished by 20% in the frontal cortex of GBA mutation carriers [37], but no changes have been reported for no-mutation carriers, so far. However, our results show diminished GBA mRNA levels in PDD caudate nucleus and in both DLB cortex and caudate nucleus independently on the mutation status suggesting that in these cases GCase deficiency already starts at the transcriptional level.

Recently, the age-related decline of GCase activity in the ageing brain has been also found. Correspondingly, it has been proposed that this diminished related to aging may act as a predisposing factor for AS accumulation and PD [38]. Since our control group included aged individuals, such changes could not be detected in the present study.

PD patients whose brains had been included in our study presented disease for at least 8 years and no *GBA* isoforms expression changes were found in the pons. Growing evidence suggests that caudal brainstem structures are involved in PD pathology even before development of nigrostriatal pathology [20]. Those early changes would include the degeneration of nondopaminergic pathways in the pons [39] indicating that molecular changes in this brain area are to be expected at very early stages of the disease. Therefore, the study of prodromal PD cases is necessary to find out whether GBA isoform expression changes are present before clinical manifestations appear.

Upon comparison with our earlier findings, the pDLB group analyzed here represents the molecular subgroup of DLB that is characterized by the drastic diminution of two main beta-synuclein (BS) gene (*SNCB*) transcripts in the cerebral cortex [20]. The lack of this natural AS antiaggregants [40, 41, 42] would strongly enhance AS oligomerization and aggregation in the cortex of these brains. It has been also shown that diminished GCase levels increase AS aggregation rate [43]. The joint effect of the drastic decrease of two key proteins primarily involved in maintaining soluble and functional AS could be the main cause of disease development in this DLB subgroup with pure LBP in the brain and a short and aggressive disease course.

On the other hand, in cDLB the temporal cortex shows diminution of only one of the two *SNCB* transcripts [20] and of only one *GBA* transcript. The remaining BS and GCase would be sufficient to avoid reaching high AS aggregation rates, so that in cDLB additional factors enhancing AS aggregation must participate to achieve a similar pathological effect to pDLB. Indeed, LBP is accompanied by characteristic AD changes in cDLB brains, a fact that suggests the involvement of elements other than AS-aggregation factors.

The caudate nucleus in PDD showed diminution of more than a half of GBA1 levels, including GBA1tv1 and GBA1tv5 that is accompanied by SNCB over-expression in that brain area [20]. Taken together, these findings indicate that different mechanisms trigger the AS aggregation process in the various neurological conditions displaying LBP.

### Expression of GBA transcripts in blood

We also analyzed expression of all three GBA transcripts in blood obtained from DLB and PD patients. In contrast to our findings in brain, in blood only GBAtv1 exhibited expression changes corresponding to significant diminution in both DLB and PD. These results correlate with those of Alcalay and colleagues who measured GCase activity in blood of PD patients with and without *GBA* mutations, and as the major finding they observed lower GCase enzyme activity in PD patients [14]. Our results of diminished GBA mRNA in blood of LBD patients suggest that here GCase deficiency starts at the transcriptional level and could represent the peripheral response to the diminution of GCase activity in the brain.

When we further studied the impact of disease duration from disease onset and age of disease onset on GBA expression, we detected the linear correlation between disease onset from duration and GBAtv1 expression in PD. The shorter the duration from disease onset the lower GBAtv1 levels which became normal with disease progression of more than 6 years. These results must be further explored to determine if GBAtv1 mRNA could be a valid biomarker for LBD. During the past years, the determination of mRNA expression changes in blood has been established as valid biomarker for disease diagnosis and progression, including PD [44]. In this context, different mRNAs have been proposed as biomarkers to identify or monitor PD patients [45-47], and GBAtv1 should be evaluated as useful part of a corresponding diagnostic panel.

When GBAtv1 expression was analyzed by age at onset, both DLB and PD showed linear correlation between GBAtv1 expression and disease onset, but with opposite tendencies. Whereas in DLB lowest GBAtv1 levels were detected in patients with earliest onset, in PD these were seen for patients with latest onset. Age at onset plays an important role for disease progression and has been postulated to be also opposite for DLB and PD. Whereas disease progresses more slowly in patients with young-onset PD, DLB shows a more aggressive course in patients with early onset [48-49].

An additional challenge in the clinical practice is the differential diagnosis of DLB versus AD due to multiple overlapping features, especially at early disease stages [50, 51]. Although diagnostic criteria for DLB have been improved substantially over the past years [52], its differential diagnosis versus AD remains very difficult. To address this difficulty, the specific diminution of GBAtv1 in early-onset as well as early-stage DLB could be applied as biomarker to differentiate between DLB and AD. The availability of reliable biomarkers is an urgent need to enhance diagnostic competency of dementias in the clinical practice.

While promising, the results obtained in the present study must be affirmed in other populations. Furthermore, larger patient groups must be also analyzed to confirm decreased GBA mRNA in blood of DLB and PD patients. Specifically, the suitability of GBAtv1 mRNA as early diagnostic biomarker needs to be studied in blood of AD patients.

In conclusion, in brain we identified disease group specific expression profiles of GBA transcripts in the temporal cortex of DLB and the caudate nucleus of PD. Dysregulation of alternative splicing was detected in brain but not in blood, where GBAtv2 and GBAtv5 did not show altered expression. The analysis of GBAtv1 expression in blood revealed its diminution in LBD, but especially in patients with early onset DLB and in patients with early PD. Finally, an opposite tendency of GBAtv1 expression levels was found in DLB and PD when analyzed in dependency on the age of disease onset with lowest levels in youngest DLB but oldest PD patients.
